# Long-Term Alterations of Cytokines and Growth Factors Expression in Irradiated Tissues and Relation with Histological Severity Scoring

**DOI:** 10.1371/journal.pone.0029399

**Published:** 2011-12-22

**Authors:** Patrice Gallet, Bérengère Phulpin, Jean-Louis Merlin, Agnès Leroux, Pierre Bravetti, Hinda Mecellem, Nguyen Tran, Gilles Dolivet

**Affiliations:** 1 EA4421 SiGReTO Nancy University, Faculty of Medicine, Vandoeuvre-lès-Nancy, France; 2 Head and Neck Surgery and Dental Units, Oncologic Surgery Department, Centre Alexis Vautrin, Vandoeuvre-lès-Nancy, France; 3 Pathology and Tumor Biology Department, Centre Alexis Vautrin, Vandoeuvre-lès- Nancy, France; 4 Oral surgery department, Faculty of Dentistry, Nancy University, Nancy, France; 5 Radiotherapy Department, Centre Alexis Vautrin, Vandoeuvre-lès-Nancy, France; 6 School of Surgery, INSERM U961, Faculty of Medicine, Nancy University, Vandoeuvre-lès-Nancy, France; 7 INSERM U961, Faculty of Medicine, Nancy University, Vandoeuvre-lès-Nancy, France; Karolinska Institutet, Sweden

## Abstract

**Purpose:**

Beside its efficacy in cancer treatment, radiotherapy induces degeneration of healthy tissues within the irradiated area. The aim of this study was to analyze the variations of proinflammatory (IL-1α, IL-2, IL-6, TNF-α, IFN-γ), profibrotic (TGF-β1), proangiogneic (VEGF) and stem cell mobilizing (GM-CSF) cytokines and growth factors in an animal model of radiation-induced tissue degeneration.

**Materials and Methods:**

24 rats were irradiated unilaterally on the hindlimb at a monodose of 30 Gy. Six weeks (n = 8), 6 months (n = 8) and 1 year (n = 8) after irradiation the mediators expression in skin and muscle were analyzed using Western blot and the Bio-Plex® protein array (BPA) technology. Additional histological severity for fibrosis, inflammation, vascularity and cellularity alterations scoring was defined from histology and immnunohistochemistry analyses.

**Results:**

A significant increase of histological severity scoring was found in irradiated tissue. Skin tissues were more radio-sensitive than muscle. A high level of TGF-β1 expression was found throughout the study and a significant relation was evidenced between TGF-β1 expression and fibrosis scoring. Irradiated tissue showed a chronic inflammation (IL-2 and TNF-α significantly increased). Moreover a persistent expression of GM-CSF and VEGF was found in all irradiated tissues. The vascular score was related to TGF-β1 expression and the cellular alterations score was significantly related with the level of IL-2, VEGF and GM-CSF.

**Conclusion:**

The results achieved in the present study underline the complexity and multiplicity of radio-induced alterations of cytokine network. It offers many perspectives of development, for the comprehension of the mechanisms of late injuries or for the histological and molecular evaluation of the mode of action and the efficacy of rehabilitation techniques.

## Introduction

Radiotherapy is an integral part of overall cancer therapy and nearly two thirds of all cancer patients have received radiotherapy at some point during their disease management [1], [2]. Its efficacy is still limited by the tolerance of healthy tissue included in the target volume of irradiation and by its side effects. Understanding the mechanisms of radiation-induced tissue degeneration is therefore essential to improve the tolerance of healthy tissues to radiation and to develop methods of tissue rehabilitation.

Histologically, the four main phenomena involved in late effects development appear to be inflammation, fibrosis, vascular alterations and cellular depletion [3], [4], [5], [6]. The involvement of each of these phenomena in the genesis of late effects is highly debated.

After radiotherapy, two types of deterministic effects are classically distinguished depending on their time of occurrence: early effects and late effects. This is based on clinical and on physiopathological concepts: early effects are easily predictable and occur soon after irradiation. Late effects can appear clinically months, even years after exposure to ionizing radiation [7], [8]. Nevertheless, it should be noted that the distinction between acute and late effects is arbitrary because it is actually a continuum [3], [4]. Late effects are the consequences of an imperfect tissue remodeling and of persistent radiation induced injuries. However, the molecular mechanisms involved in the development of these effects remain unclear. Chronic radio-induced toxicity may be induced by dysregulation of many mediators including perpetual cascades of cytokines [4], [5], [7], [9], [10]. In the current physiopathological models of radiation-induced tissue degeneration hypoxia plays a key role [11]. It perpetuates cellular damages, so that normal tissue regeneration is impossible. However, most of the studies were focused on lungs [12], , and the precise role of hypoxia in other tissues is still unclear.

Indeed most clinical prospective studies assay growth factors and interleukins on serum in order to predict symptomatic radio-induced lung injury [15], [16], [17], [18], [19].

Within tissues, intercellular communication pathways are complex and include autocrine and paracrine mechanisms. Therefore, the resulting effect depends on the local balance between different types of mediators whose regulation and spatio-temporal distribution are crucial [20], [21]. The balance cannot be understood without studying the local equilibrium. Consequently, the development of animal model of radio-induced injury is a necessity. Some studies have investigated the tissue concentrations of cytokines and interleukins, mainly in lungs [12], [13], [14], [22], gut [23], [24], or brain [25] but studies on skin and muscle are rare. In addition, all these studies are focused on one or at most 3 mediators [13], [14], [22], [23], [24], [25], [26].

**Figure 1:**
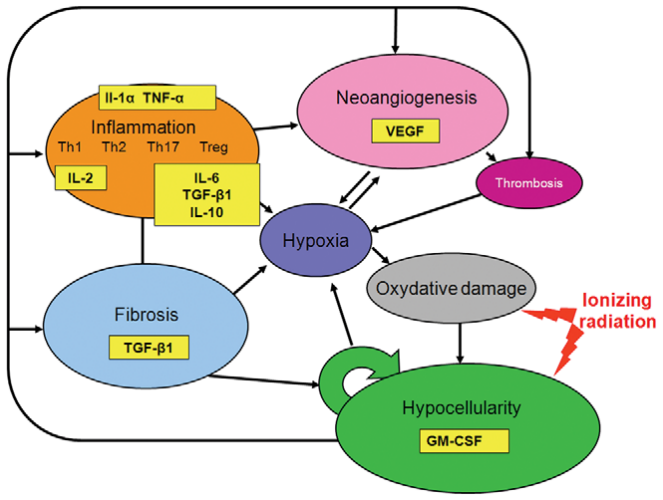
Simplified model of the complex network of interacting processes and signals in the pathogenesis of radio-induced injury. The main mediators of the four cytokine and growth factor families involved in inter-cellular exchanges during radiation-induced tissue degeneration in skin and muscles are.represented, namely the expression of proinflammatory (IL-1α, IL-2, IL-6, TNF-α, IFN-γ), profibrotic (TGF-β1), proangiogenic (VEGF) and stem cell mobilizing (GM-CSF) mediators.

The aim of this study was to analyze the variations of the main mediators of the four cytokine and growth factor families involved in inter-cellular exchanges during radiation-induced tissue degeneration in skin and muscles. An animal model of radiation-induced tissue degeneration was used for this purpose [27]. The expression of proinflammatory (IL-1α, IL-2, IL-6, TNF-α, IFN-γ), profibrotic (TGF-β1), proangiogenic (VEGF) and stem cell mobilizing (GM-CSF) cytokines (Figure 1) was analyzed using Western blot and the Bio-Plex® protein array (BPA) technology. Additional histology and immnunochemistry studies were performed.

## Materials and Methods

### Ethics Statement

The experimental protocol was conducted in accordance with the regulations of our local ethics committee (The Ethics Committee of Nancy Lorraine-France, approval authorization number: C54-547-5) and with the Animal Welfare Act of the National Institutes of Health Guide for the Care and Use of Laboratory Animals (NIH Publication no. 85-23, Revised 1996).

### Animals

This 12-month study was conducted using 24 adult male Wistar rats (Janvier CERJ, Le Genest Saint Isle, France) with an initial body weight of 420–460 g. The rats were maintained in a specific environment with controlled temperature and humidity and an automatically regulated 12-h light/dark cycle, and were fed a standard commercial diet and given water *ad libitum*.

### General experimental design

Under general anesthesia, the animals (3 groups of 8 rats) were administered a single dose of 30 Gy unilaterally on the hindlimb. The first group was euthanized 6 weeks, the second six months and the third one year after irradiation. Immediately before sacrifice, tissue samples of skin and muscle were collected on each hindlimb in order to process BPA assay and western blot. The remaining tissues were removed and fixed for histological and immunohistochemical analysis. Unirradiated hindlimbs were used as controls.

### Irradiation procedures

Cobalt 60 irradiation was chosen to minimize the difference between the absorbed dose in soft and hard tissues. Prior to irradiation, a scanner acquisition (Philips Brillance 40) enabled the calculation of dosimetry (Isogray 3.0 software Dosisoft). Irradiation of the hindlimb was performed under general anesthesia as described previously (21). Briefly, the animals were placed in a prone position upon a thick polystyrene phantom. The hindlimbs were additionally immobilized by adhesive tape. The skin distance focus was 70 cm, and the field size was 20x30 cm. The lead collimating block was positioned on a 0.5-cm thick acrylic platform to shield the body, allowing exposure of only the hindlimb without the pelvis. Radiation was delivered in a vertical beam from a Theratron® 780CX-ray machine delivering γ-rays of 1.25 MeVenergy. The irradiated volume was 40 cm3 at a dose rate of 1.4 Gy/min. The room temperature during irradiation was 22°C.

### Histopathological and immunochemistry studies

Gastrocnemius muscle and skin were removed and fixed in AFA (acid acetic, formaldehyde and alcohol). Each sample was embedded in paraffin, cut into 5-µm sections and stained prior to light microscopic observation: hematoxylin eosin, sirius red and FVIII immunohistochemistry (polyclonal rabbit, 1∶400, Dako Corporation, Trappes, France) were performed on Ventana Benchmark.

All microscope images (microscope Aristopla, Leica®), were captured with a camera (Olympus DP Controler, Olympus Optical) and analyzed with image analyzing software (TRIBVN-ics image communication software version 1.5, Chantillon France).

### Assessment of the severity of lesions

**Figure 2:**
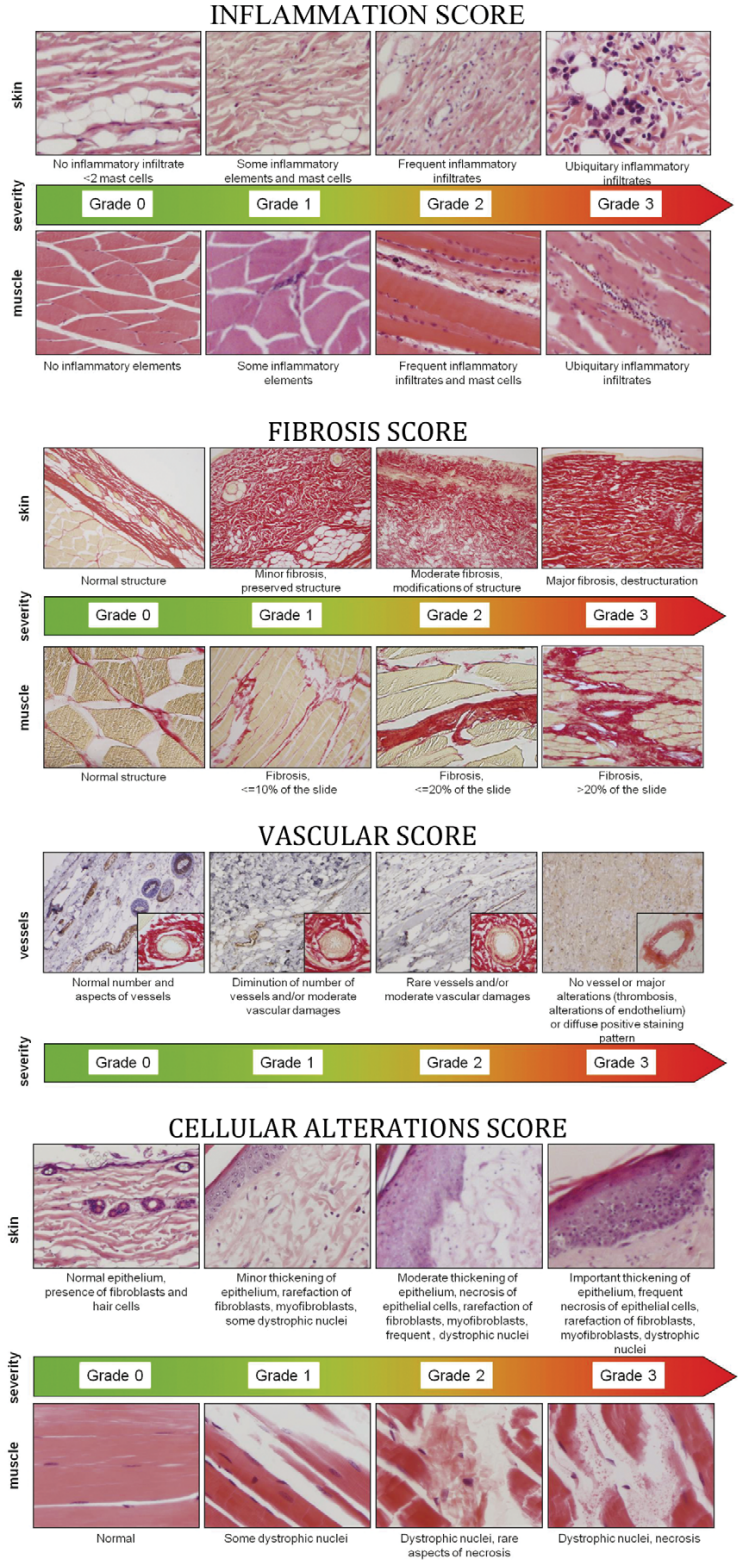
Scale of severity for inflammation, fibrosis, vascularity and cellular alterations. The inflammation score, evaluated on hematoxylin eosin-stained slides, was based on the presence of inflammatory infiltrates. The fibrosis score evaluated on sirius red-stained slides, depend on the intensity of the fibrosis and the modification of the tissular structure. The vascular score was evaluated both on sirius red- and FVIII-stained slides. It was calculated from the number and the alterations of structure of vessels. The cellular alterations score was evaluated on hematoxylin eosin-stained slides. In skin, it was calculated according to the loss and necrosis of cells as well as the number of myofibroblasts. In muscles, it depends mainly on the presence of dystrophic nuclei and necrosis.

Histopahological analyses were performed in skin and muscles specimens taken from irradiated and control unirradiated hindlimb. The four following parameters were studied: fibrosis, inflammatory infiltrates, vascular alterations, cellular depletions and alterations. For each parameter, a score of severity was established, from 0 (no alteration) to 3 (severe alterations), using the visual scale presented here (Figure 2).

Each captured image was blindly analyzed twice at three weeks interval by two independent observers. Intra and inter observer reliability of the classification was verified by the concordance with the Kappa test, with a linear ponderation for variables with several categories.

### Protein extraction

Protein from tissue specimens were extracted by using a cell lysing kit (Bio-Rad) according to the manufacturer's recommendations. The specimens were disrupted using a steel-bead tissue lyser (Tissuelyser, Qiagen) for 3 min at 30 cycles per sec. Protein concentration in supernatants was determined using the BioRad Dc Protein assay according to the manufacturer's recommendations. Then, the proteins extracts were stored frozen at −80°C until analyzed.

### BPA assay

Multiplex sandwich bead immunoassays were used to analyze the expression of the cellular mediators. All experiments were carried out in triplicate. Protein concentration was determined as indicated above, adjusted to 1 µg/ml of total protein. Protein extracts were transferred into 96-well dishes and diluted with buffered solution (Lincoplex assay buffer). Fluorescent capturing beads coupled to antibodies directed against IL-1α, IL-2, IL-6, IL-10, TNF-α, IFN-γ, VEGF and GMCSF (Linco Research (R-CYTO-80K-08)) were mixed. The antibody-conjugated beads were added into each well and incubated for 12 h at 4°C. The plates were washed (Lincoplex wash buffer) and incubated (1h at 21°C) with biotinylated antibodies to fix each target protein. The plates were then washed and Streptavidin–phycoerythrin solution was then added (30 min at 21°C).

Results were recorded as mean fluorescence intensities and normalized to the data measured in the positive controls.

Non-irradiated limbs were used as controls and all data were analyzed respectively to control at the same time. Skin and muscles were analyzed independently.

### Western blot analysis

Protein concentration was determined as indicated above, adjusted a concentration of 12 µg/ml of total protein. Dilution was performed with buffer Laemmli (Bio-Rad®). TGF-β1 activation was performed by heating the specimen at 80°C for 5 min. The proteins were electrophoresed at 100 V for 120 min (PowerPac 200, Bio-Rad) in SDS-polyacrylamide gel (10%) and then transferred onto a polyvinylidene difluoride membrane (Hybon-P–GE Healthcare Amersham® ) at 80 mA for 30 min by use of a Transblot SD 5Bio-Rad). Nonspecific binding was blocked with 5% milk for 1 h at room temperature. Immunodetection of the proteins was performed with specific primary antibody (TGF-β dilution range 1/500 sc-52893, Santa Cruz Biotechnology) in TBST 0.1% with 5% milk and incubated overnight at 4°C. The membranes were washed and incubated with mouse peroxydase-secondary anti-IgG1 (dilution range 1/2000) for 1 h and washed. Immunoreactive proteins were visualized by using electroluminescent reagent (Kit ECL Western blotting Detection reagents–GE Healthcare Amersham ®).

Each dried blot was numerized at 100 dpi (Perfection 1670 scanner; Epson) and processed using ImageJ, Java-based image processing software (Rasband WS; NIH).

The area of the peak was determined and compared to that of a reference sample of known concentration in TGF-β1. Values were expressed as percentage of control.

Non-irradiated limbs were used as controls and all data were analyzed respectively to control at the same time. Skin and muscles were analyzed independently.

### Comparison of histopathological and immunochemistry studies with BPA assays and western blot

For each severity score of fibrosis, inflammation, vascular alterations, cellular depletions and alterations were reported to corresponding levels of mediators expression.

### Statistical analysis

The statistical significance of differences between the groups was determined using the Student's t-test. For each score of severity, the correlations between the score and the level of expression of each mediator were determined. p<0.05 was considered as significant.

## Results

### Histopathological results

**Figure 3:**
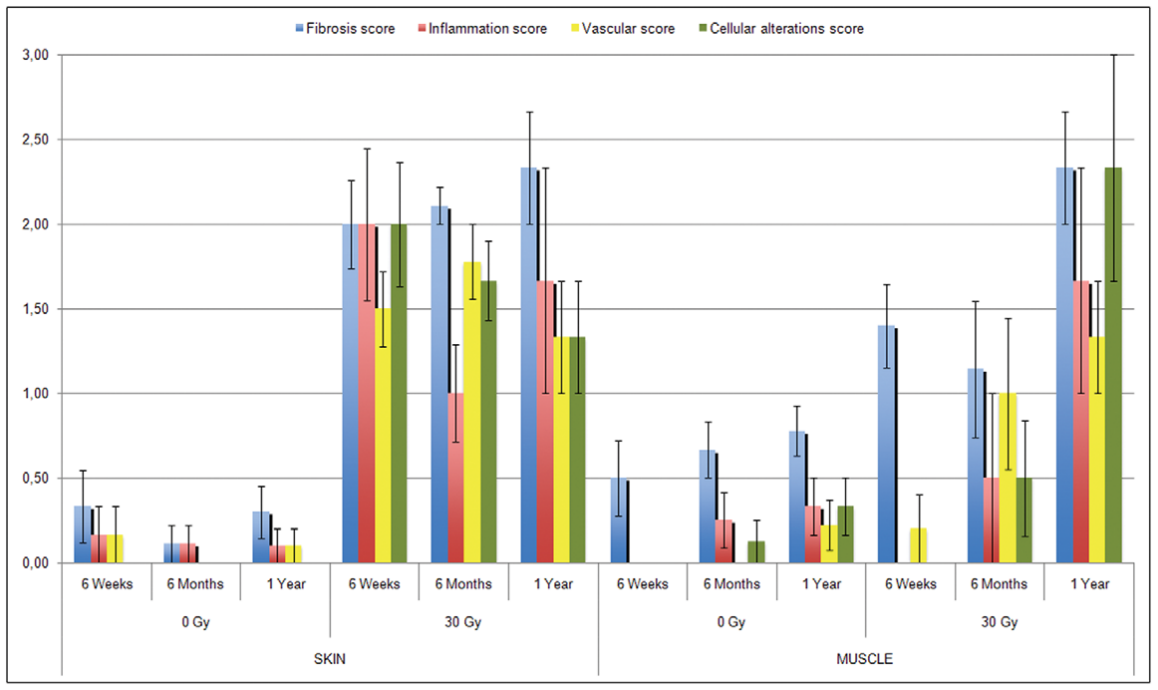
Evolution of the score of fibrosis, inflammation, vascularity and cellular alterations with time in skin and muscle. The fibrosis score showed an increased with time in both skin and muscle. In skin, the inflammation score appeared to decrease at 6 months, but increased at 1 year. In muscle, the inflammation score increased consistently with time.

Irradiation at a monodose of 30 Gy induced in all irradiated tissue a marked increase of histological scoring compared to unirradiated hindlimb. However, no significant differences could be found over time (Figure 3).

An increase in fibrosis score was observed 1 year after irradiation, in both skin and muscle. In skin, the inflammation score appeared to decrease at 6 months, but increased at 1 year. However, in the muscle, the inflammation score increased with time.

Inter-observer reproducibility was good for all the parameters of the histological classification (Kappa value are listed Table 1).

**Table 1:** Intra- and inter- observer reproducibility of the histological scoring in skin and muscle. All figures are mean Kappa values +/− standard deviations.

	Intra-observers reproducibility		Inter-observers reproducibility
	A	B	
***Skin***			
Fibrosis score	0.78+/−0.09	0.73+/−0.1	**0.69**+/−**0.1**
Inflammation score	0.82+/−0.08	0.76+/−0.09	**0.77**+/−**0.09**
Vascular score	0.41+/−0.11	0.69+/−0.1	**0.77**+/−**0.09**
Hypocellularity score	0.78+/−0.09	0.77+/−0.09	**0.73**+/−**0.09**
***Muscle***			
Fibrosis score	0.44+/−0.16	0.59+/−0.12	**0.67**+/−**0.12**
Inflammation score	0.54+/−0.14	0.65+/−0.14	**0.86**+/−**0.12**
Vascular score	0.77+/−0.14	0.47+/−0.14	**0.82**+/−**0.11**
Hypocellularity score	0.62+/−0.14	0.47+/−0.14	**0.76**+/−**0.16**

### Expression of cytokines and growth factors

#### Cytokine expression in irradiated skin

**Figure 4:**
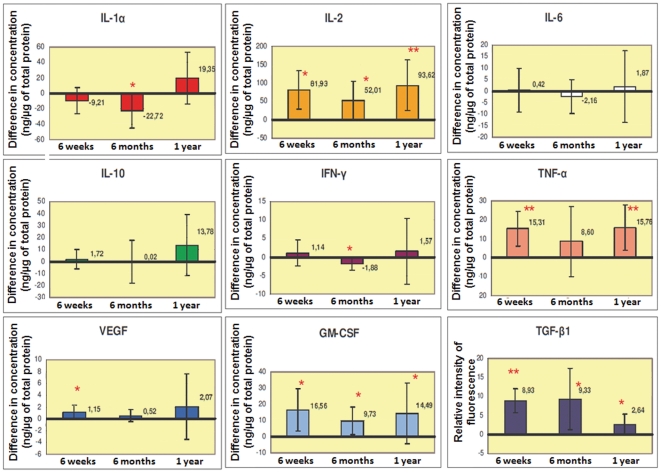
Mean differences of expression of different mediators between healthy skin and irritated skin over time. All pro-inflammatory mediators (IL-1α, TNF-α, IL-2, IFN-γ, IL-6), the pro-angiogenic mediator VEGF, and the stem cell-mobilizing cytokine GM-CSF evolved consistently with time, the level of expression measured at 6 weeks appeared to decrease at 6 months, then the trend reversed at 1 year. The expression of the profibrotic mediator TGF-β remained markedly increased during the study. (*: p<0.05 and **: p<0.01)

During this study, the control values obtained from the unirradiated contralateral hindlimb were stable over time. The results are shown in Figure 4.

All pro-inflammatory mediators (IL-1α, TNF-α, IL-2, IFN-γ, IL-6) evolved consistently with time. Six weeks after irradiation, TNF-α was significantly higher than control (+66%, p = 0.009) as well as IL-2 (+458%; p = 0.013). The expression level of IFN-γ also tended to rise. Six months after irradiation, the inflammatory reaction tended to attenuate: IL2 remained moderately elevated (+83%, p = 0.028) but the increase of TNF-α was no longer significant (+22%, p = 0.231) while IFN-γ and IL-1α decreased markedly (respectively −34%, p = 0.035 and −42%, p = 0.015). However, one year after irradiation, there was a small intensification of inflammatory reaction, with a significant increase of TNF-α (+53%, p = 0.002), and IL-2 (+350%, p = 0.006). At one year, the expression level of IL-1 was also found to be increased but did not reach statistical significance. During the study, the difference in the expression of IL-6 and IL-10 between irradiated hindlimbs and non irradiated hindlimbs never reached statistical significance. To summarize, the expression of all proinflammatory cytokines (IL-1α, TNF-α, IL-2, IFN-γ, IL-6) measured at 6 weeks appeared to decrease at 6 months, then the trend reversed at 1 year.

Positive correlations were found between TNF-α and IL-1α (r = 0.690, p<0.001), IL-2 and TNF-α (r = 0.558, p<0.001) and IFN-γ and IL-1α (r = 0.429 p<0.001). As expected, the anti-inflammatory mediator IL-10 was negatively correlated to TNF-α (r = −0.324, p = 0.001) and IL-1α (r = −0.182, p = 0.05).

The expression of the profibrotic mediator TGF-β remained markedly increased during the study. Six weeks after irradiation, the level of TGF-β was 2.2 times (p = 0.001) higher than that measured on unirradiated tissue and reached 2.7 times (p = 0.013) 6 months after irradiation. At one year, the level of TGF-β was still significantly increased but to a lesser extent (+65%, p = 0.017). The TGF-β was negatively correlated to IL-10 (r = −0.252, p = 0.014).

Six weeks after irradiation, the expression level of the pro-angiogenic mediator VEGF was markedly increased (+177%, p = 0.05). This increase persisted over time and VEGF expression doubled (+119%, p = 0.181) 6 months after irradiation and reached four times the controlateral unirradiated level (+323%; p = 0.233) after one year. However the values did not reach statistical significance, probably due to the small number of sample. The pattern of VEGF expression, with a slight decrease at 6 months and an increase at one year, was similar to the pattern of expression of pro-inflammatory cytokines. A positive correlation was observed between VEGF and IL-2 (r = 0.585; p<0.001) and between VEGF and GM-CSF (r = 0.689; p<0.001).

Six weeks after irradiation, the expression of the stem cell mobilizing cytokine GM-CSF was higher than control (x33.6; p = 0.047). This increase persisted over time with an increase of 2.5 times (p = 0.022) and 5.5 times (p = 0.048) respectively 6 months and one year after irradiation. As for VEGF, the profile of evolution was similar to the inflammatory reaction and IL-2 was positively correlated with GM-CSF (r = 0.744, p<0.001).

#### Cytokine expression in irradiated muscle

Differences between irradiated and non irradiated samples of muscles were less marked and most of the results were not significant.

The evolution of the mediators was slightly different from that found in the skin. The inflammatory reaction seemed initially less intense, then appeared gradually, with a progressive trend to increase of IL-1, TNF-α, and IL-2, which was not statistically significant.

The level of TGF-β increased later than in the skin, but the differences between irradiated and non irradiated samples were very marked at 6 months (more than 10 fold increase, p = 0.017) and at 1 year (3 fold increase, p = 0.041). The profile of variation in the expression of GM-CSF and VEGF seemed to be similar to that of the inflammatory reaction.

### Relations between histopathological results and expression of mediators

**Figure 5:**
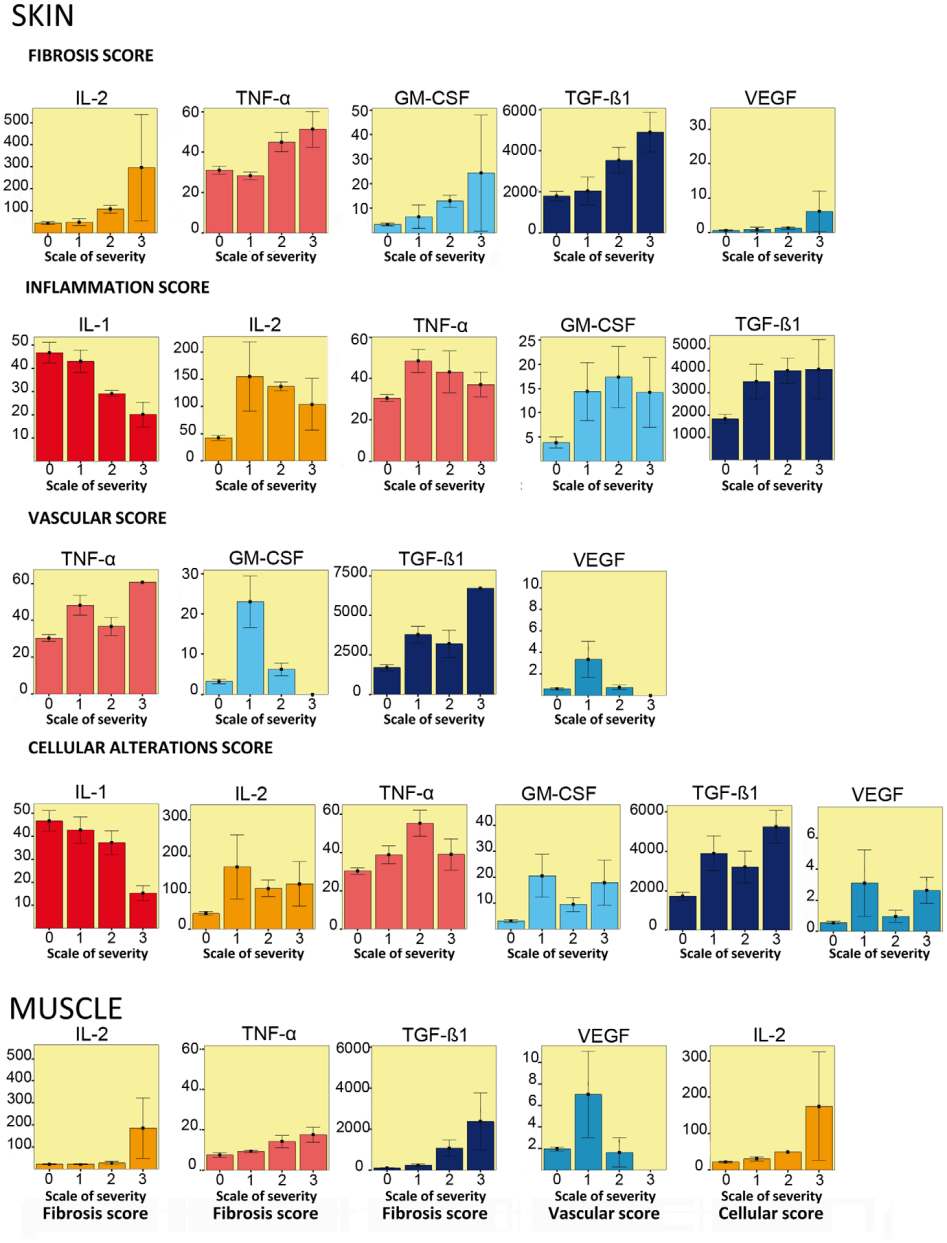
Variation of expression of cytokine and growth factor for each score of severity from inflammation, fibrosis, vascularity and cellular alterations in skin and muscles. In skin only significant variations are shown. The y-axis corresponds to the level of expression of each cytokine in ng/µg of total protein.

The significant variation of expression of cytokine or growth factor for each score of severity from inflammation, fibrosis, vascularity and cellular alterations are shown in Figure 5.

In skin, the global profile of evolution in time was similar for pro-inflammatory cytokines and for histological scores, always with a late intensification. Higher scores of inflammation were associated with higher levels of IL-2 (r = 0.274, p = 0.05), TGF-β1 (r = 0.484, p = 0.001) and GM-CSF (r = 0.358, p = 0.002). The impact of the variation of TNF-α on the inflammation score was moderate (r = 0.3, p = 0.05). On the contrary, IL-1 levels significantly decreased with increase of the inflammation score (r = −0.4, p = 0.008).

A significant relation was found between TGF-β1 expression and histological fibrosis scoring (r = 0.554, p = 0.002). The fibrosis score also increased with the level of IL-2 (0.453, p = 0.003), and with TNF-α (r = 0.497, p = 0.001). No relation could be found between the levels of expression of the other pro-and anti-inflammatory mediators (IL-1, IL-6, IL-10) and the fibrosis score (data not shown), the expression levels of GM-CSF and VEGF both increased with the severity of fibrosis (r = 0.455, p = 0.002 and r = 0.342, p = 0.027 respectively).

The levels of expression of the key mediators were not related with vascular score, except for TGF-β1 (r = 0.521, p<0.001), whose level of expression was increased with vascular alterations, and at a lower level for VEGF and GM-CSF which increased with mild alterations (p<0.05).

The cellular alterations score was significantly related with the level of IL-2 (r = 0.3, p = 0.05), VEGF (for mild alterations, p<0.05), GM-CSF (r = 0.37, p = 0.017) and TNF-α (r = 0.5, p = 0.001). Finally, the level of IL-1 expression slightly decreased when the cellular alterations score increased (r = −0.37, p = 0.02).

In muscles, the major significant relation was found between TGF-β1 expression and histological scoring with a late intensification (r = 0.67, p<0.001).

## Discussion

Radiotherapy-induced late normal tissue injury within the irradiated field is a significant cause of morbidity and decrease quality of life in cancer patients [28]. This justifies the evaluation of biomarkers predictive of late toxicity of radiation. The present study was performed in a model of late tissue radio-induced degeneration [27], enabling the assessment of histological changes and dysregulation of molecular mediators in irradiated tissues.

Despite monodose irradiation, the histological and macroscopic modifications observed in this study were very similar to those observed after radiotherapy in humans. In our model, inflammation was maximal at 30 days, followed by fibrosis starting at 6 weeks, stabilizing at 3 months but sometimes continuing through successive stages. However, the main problem with the histological analysis of irradiated tissues is that radiation-induced injuries are focal so that analysis might miss some aspects of radiation-induced degeneration. This is the reason why we developed a histological scoring system and tested its reliability prior to analysis. Intra-and inter-observers reliabilities were good, but it clearly appeared to us that histological analysis was not sufficient for precisely evaluating radio-induced tissular degeneration. In this context, the analysis of cytokine and mediator expression appeared to be an attractive method of evaluation of tissular changes after irradiation.

Very often, the analysis of expression of cytokines and growth factors is based on immunochemistry [12], [25], [26], [29] or mRNA expression analyses [23], [25], [26], [30]. This gives an insight into the production of proteins, but without quantifying their real concentration in the tissue, which is determinant for the actions of the mediators studied. In this study, the methodology was based on the direct analysis of protein using the Bio-Plex® cytokine array. Indeed, the determined levels of expression of the key mediators in this study were coherent, and the expected correlations between pro- and anti-inflammatory mediators could be identified. This confirms the reliability of the methodology.

Expression levels of the key mediators studied were consistent with the current model of radio-induced tissular degeneration.

Similarly to other works [14], [30], [31], [32], particularly in the skin, a high level of TGF-β1 was found throughout the study. TGF-β1 is known to control the regulation and inhibition of cell growth and the homeostasis of the extracellular matrix. Dysregulation of TGF-β1 is involved in the development of fibroproliferative diseases [33], [34], [35]. The increased expression of TGF-β1 was already present 6 weeks after irradiation, so that the initiation of this process was certainly earlier. Indeed, several authors found an induction of TGF-β1 during the first 24 hours after irradiation [36], [37], [38]. In this study, a significant relation was found between TGF-β1 expression and histological scoring. These results highlight the crucial role of this cytokine in the pathogenesis of radiation fibrosis.

The precise role of inflammation in the constitution of late injuries is less clear. Similarly to others studies, a chronic release of proinflammatory mediators was observed in irradiated tissues in our model. The TNF-α levels of expression were increased during all the observation period. This was consistent with other studies reporting that in the lungs, an increase of TNF-α expression was detected during the first 10 days after irradiation [13] and that values of TNF-α mRNA remained elevated 25 weeks after irradiation [23]. IL-2 levels were also elevated during all the study, with the same profile of evolution. On the contrary, IL-1α increased only later in our model. This could be surprising, as in vitro and in vivo studies highlighted that thoracic irradiation induced prolonged release of IL-1α in serum and in lung samples [30], [39], [40]. But Rube et al. already found in irradiated lung samples that after an initial increase of IL-1 during the first hours after irradiation, IL-1 returned to a baseline threshold and elevated markedly only after 8 and 16 weeks. In the present rat model, 6 weeks might correspond to the period of baseline threshold of Rube et al. study [38]. In fact, histological findings and scores were similar to these molecular changes: following an initial inflammatory phase, there was a decrease in inflammation at 6 months and then a rebound of inflammatory reaction was found at 1 year. This late intensification of inflammatory reaction was particularly marked with a very significant increase of the IL-1α expression between 6 and 12 months (p = 0.007). Similar late intensification was also observed for muscles in our model.

Chronic inflammatory reaction is usually considered as the trigger for radiation-induced fibrosis and some authors proposed that pro- and anti-inflammatory cytokines might be used as markers for predicting late injuries: Chen and coworkers [18] found that elevated pre-irradiation levels in IL-1α and IL-6 were predictive of radiation-induced symptomatic pneumopathy. Similarly, Arpin and coworkers [17] found that high levels of IL-10 were protective against radiation induced symptomatic pulmonary fibrosis after thoracic radiotherapy, while elevation of IL-6 was associated with more severe lesions. This might be explained by the known direct antagonism between IL-10 and TGF-β1, and by the inhibition of the production of IL-10 by TGF-β1 associated with IL-6 in the T-reg pathway [41]. Nevertheless, the precise role of these cytokines in the constitution of late lesions remains controversial and some studies were in contradiction with Chen's and Arpin's results. Barthelemy-Brichant and coworkers [42] found no relation between IL-6 and radiation pneumonitis and a multicytokine analysis of plasma of patients showed that only low pre-treatment levels of IL-8 were predictive of radiation pneumonitis. Our model confirms that IL-10 might play a protective role against fibrosis, as elevation of IL-10 was slightly related with lower levels of TGF-β (r = −0.25, p = 0.02), but this role is not confirmed by histological scores. Similarly, the increase of IL-1α expression was not associated with more severe lesions (fibrosis, inflammation, vascular alterations and cellular alterations scores). Finally, the relation between levels of expression of TNF-α, and the fibrosis, the inflammation or the vascular alterations scores was only moderate in our model. On the contrary, the inflammation, cellular alterations and fibrosis scores were related to variations of IL-2. Consequently, IL-2 appeared to play a crucial role in the constitution of late injuries in our model. The radio-induced injuries might be preferentially Th1-mediated. Further investigations of the immune balance would clarified the impaired interactions between Th1/Th2/Th17/Treg.

Recently, hypoxia has been placed in the center of all the models of radio-induced tissular degeneration. All authors agree on the fact that irradiation has deleterious effects on vascularization and neoangiogenesis [43], [44], [45]. VEGF is the main proangiogenic molecule and probably the most studied. Ebrahimian et al.[46] showed that irradiation hampered skin perfusion, capillary number, and VEGF plasma level. Consistent with others studies [12], [14], [25], [26], we observed an increase of VEGF and this throughout the study. VEGF usually promotes revascularization and wound healing [47], [48], but despite an increase of this angiogenic signal, histological and immunochemistral studies demonstrated typical decreased vascularity and alterations of the vascular wall [44], [45], [49]. Indeed, VEGF is known to be able to affect vessel permeability. It has been shown to induce fenestrations in capillary endothelial cells [50] and to affect occludin expression and tight junction assembly [51]. In our study, immunochemistry revealed diffuse expression of FVIII in irradiated tissues. As in the irradiated spinal cord [25], we can hypothesize that VEGF contributed to increase the vascular permeability in irradiated tissue and to perpetuate the vascular impairment.

Though we found no major relation between VEGF levels and the fibrosis score (r = 0.34, p = 0.027), the increase of VEGF we observed was more moderate than in other studies [12], [14]. This difference with existing studies might be explained by the type of tissue studied. Indeed, most of the existing studies were conducted in lung tissues, which have a particular vascularity system and hypoxia could also be the consequence of the impaired pulmonary function. The landmark of hypoxia HIF-1 is the upstream cytokine of VEGF, consequently further investigations may be necessary to link hypoxia, VEGF, vascular damage and late radiation-induced injuries in skin and muscle.

In our model, a persistent high level of GM-CSF, a cytokine with a specific ability to mobilize stem cells, was found throughout the time of the study in irradiated skin and muscle. In skin, GM-CSF appeared correlated to fibrosis score (r = 0.455, p = 0.002) and hypocellularity score (r = 0.37, p = 0.017). An increase in GM-CS had already been highlighted in irradiated lung tissue, but during the early response to radiation (<10 days) [13]. However, this increase could not be detected in serum samples after lung irradiation neither in mice [13] nor in patients [19]. As the GM-CSF level of expression was high throughout this study, it may be hypothesized that irradiated tissues try to mobilize and recruit stem cells in order to overcome the hypoxic and scarring problem [23], [52], [53], [54], [55]. The presence of stem cells in irradiated tissues might help scarring of wounded tissues [52], [53], [56]. It has been shown that injection of the growth factors G-CSF, SCF and/or GM-CSF could be used to improve radioprotection or radio-induced tissue injury [20], [23]. However, these treatments have a saturating effect [20], probably due to the limited capacity of effective recruitment of stem cells. In our model, despite the increase of GM-CSF within the irradiated tissue, histological studies showed persistent hypocellularity. The number of stem cells reaching and/or integrated in these tissues appears to be insufficient. The radio-induced alteration of blood vessel walls [45], [49], [57] and fibrosis [4], [9] probably limit the extravasation of stem cells. Consequently, stem cell therapy might provide a means to reduce radiation-induced side effects [28]. Local injection of stem cells could bring multipotent functional cells, able to replace the differentiated cells, interact with the environment through secretion of biofactors, and regulate the inflammatory microenvironment, while inducing and participating in neoangiogenesis.

### Conclusion

Our animal model of radio-induced tissular degeneration appeared to be reliable and showed a good coherence with the existing theories of the physiopathology of radio-induced degeneration. It offers many perspectives for development, for the comprehension of the mecanisms of late injuries, and for the histological and molecular evaluation of the mode of action and the efficacy of rehabilitation techniques.

The results achieved in the present study underline the complexity and multiplicity of radio-induced alterations of cytokine networks. In this context, the results achieved suggest that stem cell therapy might represent an interesting approach to overcome radiation-induced injuries, in relation with the extensive paracrine activity of stem cells.
